# Association between intrarenal venous flow Doppler and postoperative acute kidney injury in children undergoing cardiac surgery: A retrospective cohort study

**DOI:** 10.1007/s00431-025-06187-5

**Published:** 2025-06-25

**Authors:** Jin-Tae Kim, Pyoyoon Kang, Jung-Bin Park, Sang-Hwan Ji, Young-Eun Jang, Eun-Hee Kim, Hee-Soo Kim, Ji-Hyun Lee

**Affiliations:** 1 Department of Anesthesiology and Pain Medicine, Seoul National University Hospital, Seoul National University College of Medicine, # 101 Daehakro, Jongrogu, Seoul, 03080 Republic of Korea; 2 Department of Anesthesiology and Pain Medicine, Korea University Anam Hospital, Seoul, Republic of Korea

**Keywords:** Acute kidney injury, Congenital heart defect, Infant, Child, Cardiac surgical procedures, Doppler ultrasonography

## Abstract

The role of intrarenal Doppler parameters in predicting postoperative acute kidney injury (AKI) has been increasingly emphasized, but remains underexplored in children undergoing cardiac surgery. This study aimed to investigate the association between intrarenal venous Doppler patterns and the occurrence of postoperative AKI in children after congenital cardiac surgery. This retrospective study included 338 pediatric patients who underwent elective cardiac surgery between June 2019 and December 2021. Intrarenal blood flow Doppler measurements were performed before and after surgery. The primary outcome was the difference in postoperative renal venous Doppler patterns between patients with and without AKI according to the Kidney Disease: Improving Global Outcomes criteria. Multivariate logistic regression analysis was performed to identify factors associated with AKI. Of the 338 patients, 12.1% developed postoperative AKI. Patients with AKI had a higher prevalence of biphasic and monophasic renal venous flow patterns than those without AKI (63.5% vs. 19.9%, *p* < 0.001). The AKI group had higher pre- and postoperative renal resistive index than the non-AKI group. Discontinuous renal venous flow pattern, preoperative intensive care unit admission, higher Risk Adjustment for Congenital Heart Surgery-1 score and intraoperative vasoactive-inotropic score, and lower preoperative albumin levels were associated with postoperative AKI.

***Conclusion*: **Abnormal postoperative intrarenal venous Doppler patterns were associated with AKI in pediatric patients after congenital cardiac surgery. Intraoperative Doppler assessment of renal venous flow may help identify children at a higher risk of AKI, allowing for early interventions.

## Introduction

Acute kidney injury (AKI) is a common complication of major surgery. In adults, the incidence rate of postoperative AKI ranges from 8% to 64% [1–4]. In pediatric patients undergoing cardiac surgery, the incidence rate of AKI after cardiopulmonary bypass (CPB) is as high as 42% [5, 6]. Postoperative AKI is a risk factor for prolonged stay in the intensive care unit (ICU) and hospital and morbidity and mortality [7]. A previous study has reported that even an episode of mild AKI is associated with low long-term survival rates in critically ill patients [8].

Venous congestion can be caused by right heart dysfunction [9] and is a major risk factor for renal dysfunction [10]. Renal congestion can be assessed by measuring intrarenal arterial and venous flow using ultrasonography [11]. According to a recent meta-analysis, intrarenal venous pulsed-wave Doppler could help detect congestive nephropathy and predict AKI in critically ill patients [11]. Similarly, in adult patients undergoing cardiac surgery, alteration in intrarenal flow is a marker of venous congestion and is independently associated with postoperative AKI [9].

Congenital heart disease (CHD) is frequently associated with pressure and volume overload in the right side of the heart [12, 13]. which can lead to venous congestion. Right heart dysfunction remains prevalent even after congenital cardiac surgery and is highly associated with adverse clinical outcomes, such as AKI [14]. Given the critical impact of AKI on postoperative outcomes, the early identification and monitoring of at-risk patients are crucial.

We hypothesized that intrarenal blood flow, especially renal venous flow measured after congenital cardiac surgery, could be associated with postoperative AKI in children. However, no study has assessed the association between indices of intrarenal blood flow, including renal venous Doppler pattern and resistive index (RI), and AKI occurrence following cardiac surgery in pediatric patients. Therefore, this study aimed to compare intrarenal blood Doppler parameters in children with and without postoperative AKI and evaluate the association between these parameters and AKI occurrence.

## Materials and methods

### Study population

This retrospective single-center study was approved by the Institutional Review Board (IRB) of Seoul National University Hospital (IRB no. 2310-036-1473, Chairperson Professor Ok-Joo Kim, date of approval: 2023 November 11), and the need for informed patient consent was waived. This study was conducted in accordance with the Declaration of Helsinki. We reviewed the electronic medical records of pediatric patients aged <18 years who underwent cardiothoracic surgery between June 2019 and December 2021. We searched for patients who underwent elective open cardiac surgery with or without CPB at Seoul National University Children’s Hospital. Surgeries associated with extracorporeal membrane oxygenation (ECMO) or pacemakers and those involving postoperative bleeding or wounds were excluded. Of these patients, only those with renal vein Doppler data measured by an anesthesiologist before and after CPB were included in the analysis.

### Data collection and definitions

Through an electronic medical record review, patients’ baseline characteristics and preoperative, intraoperative, and postoperative variables were collected. Surgery was categorized using the Risk Adjustment for Congenital Heart Surgery-1 (RACHS-1). Preoperative variables included a history of pulmonary hypertension, admission to the ICU or mechanical ventilator care, and preoperative laboratory values, such as hemoglobin concentration, plasma albumin level, glomerular filtration rate (GFR), creatinine level, and fibrinogen concentration. The intraoperative variables included anesthetic duration, CPB time, aortic cross-clamp time, amount of transfused blood, infused plasma or colloid volume, urine output, and vasoactive-inotropic score (VIS). The VIS was calculated as follows: dopamine dose (μg/kg/min)+dobutamine dose (μg/kg/min)+100×epinephrine dose (μg/kg/min)+10×milrinone dose (μg/kg/min)+10,000×vasopressin dose (U/kg/min)+100×norepinephrine dose (μg/kg/min). Transesophageal echocardiographic data obtained before and after the operative procedure were included as intraoperative variables. Postoperative outcome variables included the duration of mechanical ventilation, length of ICU and hospital stay, AKI, and other major adverse events (MAEs). MAEs included cardiac arrest, re‐sternotomy due to hemodynamic instability, ECMO support, significant arrhythmia requiring treatment, cerebral hemorrhage or infarction, and pulmonary complications [15]. Postoperative day (POD) 0 was defined as the period from postoperative ICU admission to midnight on the day of surgery, and PODs 1–7 were defined as sequential 24-h intervals thereafter.

AKI occurrence was determined according to the Kidney Disease: Improving Global Outcomes (KDIGO) AKI criteria, which classifies AKI into three stages based on changes in serum creatinine. AKI staging was determined as follows: grade 1, increase in plasma creatinine of 0.3 mg/dL (>48 h) or increase in plasma creatinine up to 150–200% of baseline level (>7 days); grade 2, increase in plasma creatinine to 200–300% of baseline level; grade 3, increase in plasma creatinine to >300% (>threefold) of baseline level or estimated glomerular filtration rate ≤35 mL/min per 1.73 m^2^, or a need for postoperative renal replacement therapy [16]. Urine output criteria were not used due to incomplete documentation in our dataset. Baseline creatinine was defined as the lowest serum creatinine value measured within the three months prior to surgery or minimum values of serum creatinine measured within 7 days after admission [17].

### Anesthesia protocol

All patients received routine anesthesia induction with atropine (0.02 mg/kg), thiopental sodium (3–5 mg/kg) or midazolam (0.1 mg/kg), fentanyl (1–5 mcg/kg), and rocuronium (1 mg/kg). After endotracheal intubation, mechanical ventilation was commenced and controlled to obtain a partial pressure of carbon dioxide of approximately 40 mmHg using the volume-controlled mode with a tidal volume of 7–8 mL/kg and a positive end-expiratory pressure between 5 and 7 cmH_2_O during surgery. Anesthesia was maintained using sevoflurane and a continuous infusion of remifentanil, midazolam, and rocuronium. Monitoring included arterial and central blood pressure and regional cerebral oxygen saturation.

### Echocardiographic variables

Generally, at our center, immediately after anesthesia induction and before the commencement of surgery, transthoracic echocardiography (TTE) was performed and stored as preoperative data. Subsequently, a transesophageal echocardiography (TEE) probe was inserted, and continuous echocardiographic monitoring was maintained until the end of surgery. In this study, we collected TTE data before the start of surgery and TEE data at the end of the procedure. All echocardiographic data were measured by two expert echocardiographers (K.J.T. and L.J.H.), each with >12 years of echocardiography experience. The echocardiography systems used were Philips iE33 (Philips Healthcare, Andover, MA, USA) and Vivid E95 echocardiography systems (Wauwatosa, WI, USA).

The data included the ejection fraction of the systemic ventricle assessed using Simpson’s method as the systolic function, intrarenal venous flow pattern measured at the interlobar renal vein, and the RI of the renal artery. Intrarenal blood flow was assessed by first obtaining an image of the renal parenchyma and then setting the velocity range of the color Doppler to approximately 16 cm/s to identify the interlobar artery and vein. The sample volume was then positioned, and pulsed-wave Doppler was performed. Recordings were obtained over three consecutive cardiac cycles. Preoperative measurements were obtained with the patient in the supine position using a sector transducer with a frequency range of 1–6, 3–8, or 4–12 MHz depending on the size of the child. For TEE, the probe was placed in the gastric position to locate the kidneys.

**Fig. 1:**
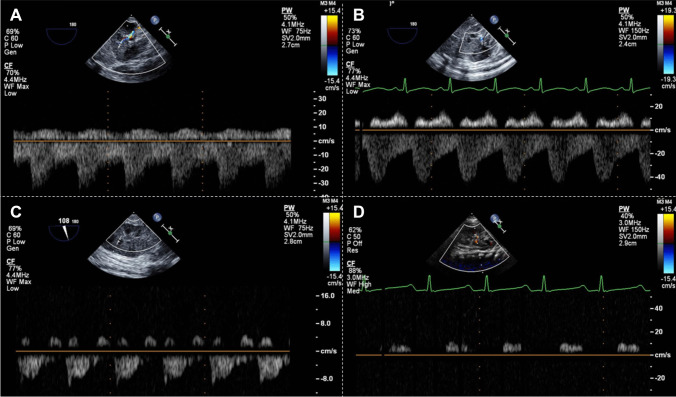
Four types of intrarenal venous flow Doppler waveform assessed via transesophageal echocardiography. **A**, continuous flow; **B**, pulsatile flow; **C**, biphasic flow; **D**, monophasic flow. Patterns **A** and **B** were classified as pattern 1, **C** as pattern 2, and **D** as pattern 3

The intrarenal venous flow patterns were categorized into four patterns, which were previously described: continuous flow, pulsatile flow (with brief interruption of venous flow during atrial contraction), biphasic flow (with venous peaks during systole and diastole), and monophasic flow (with venous peaks during diastole) (Fig. 1) [9]. The renal RI was calculated as the difference between the highest systolic blood velocity (Vmax) and the lowest diastolic blood velocity (Vmin) divided by the highest systolic blood velocity ([Vmax−Vmin]/Vmax). The arterial velocity was assessed more than three times, and the RI was calculated as the average of all values.

### Statistical analyses

The primary outcome was the difference in renal vein Doppler pattern between patients with and without AKI. The secondary outcomes were as follows: differences in intraoperative variables, including other echocardiographic measurements, and postoperative outcome variables between patients who developed postoperative AKI and those who did not and the predictive power of renal vein Doppler patterns for postoperative AKI. Categorical variables were evaluated using the chi-squared test. In the case of RACHS-1, the chi-squared test was performed by dividing categories into 1–2 as low grade and 3–6 as high grade. The intrarenal venous blood flow was classified into three patterns. Pattern 1 is a continuous flow or brief interruption of the flow, which is considered normal. Pattern 2 is a biphasic flow, and pattern 3 is a monophasic flow detectable only during diastole. Spearman’s rank correlation was performed to evaluate the relationship between the renal venous Doppler pattern and AKI grade using the KDIGO criteria.

We used multivariate logistic regression analysis to determine whether renal venous Doppler patterns and other factors were associated with postoperative AKI on POD 7. First, we identified the factors related to AKI using univariate analysis, and multivariate stepwise selection logistic regression was then performed using factors with a *p* value ≤0.1. Before conducting the logistic regression, we assessed multicollinearity among independent variables by calculating variance of inflation factors (VIFs), and confirmed that VIF values were below 10. The Hosmer–Lemeshow goodness‐of‐fit test was used to compare the estimated‐to observed likelihood of outcome. Model discrimination was evaluated using C-statistics.

All data are expressed as means±standard deviations or medians (interquartile ranges), unless otherwise specified, after the normal distribution was tested using the Shapiro–Wilk test. The continuous variables of the study population were evaluated using the independent *t*-test or Mann–Whitney *U* test. All statistical analyses were performed using the SPSS (SPSS 26.0; IBM Inc., Armonk, NY, USA) software, and a *p* value <0.05 was considered statistically significant.

## Results

**Fig. 2:**
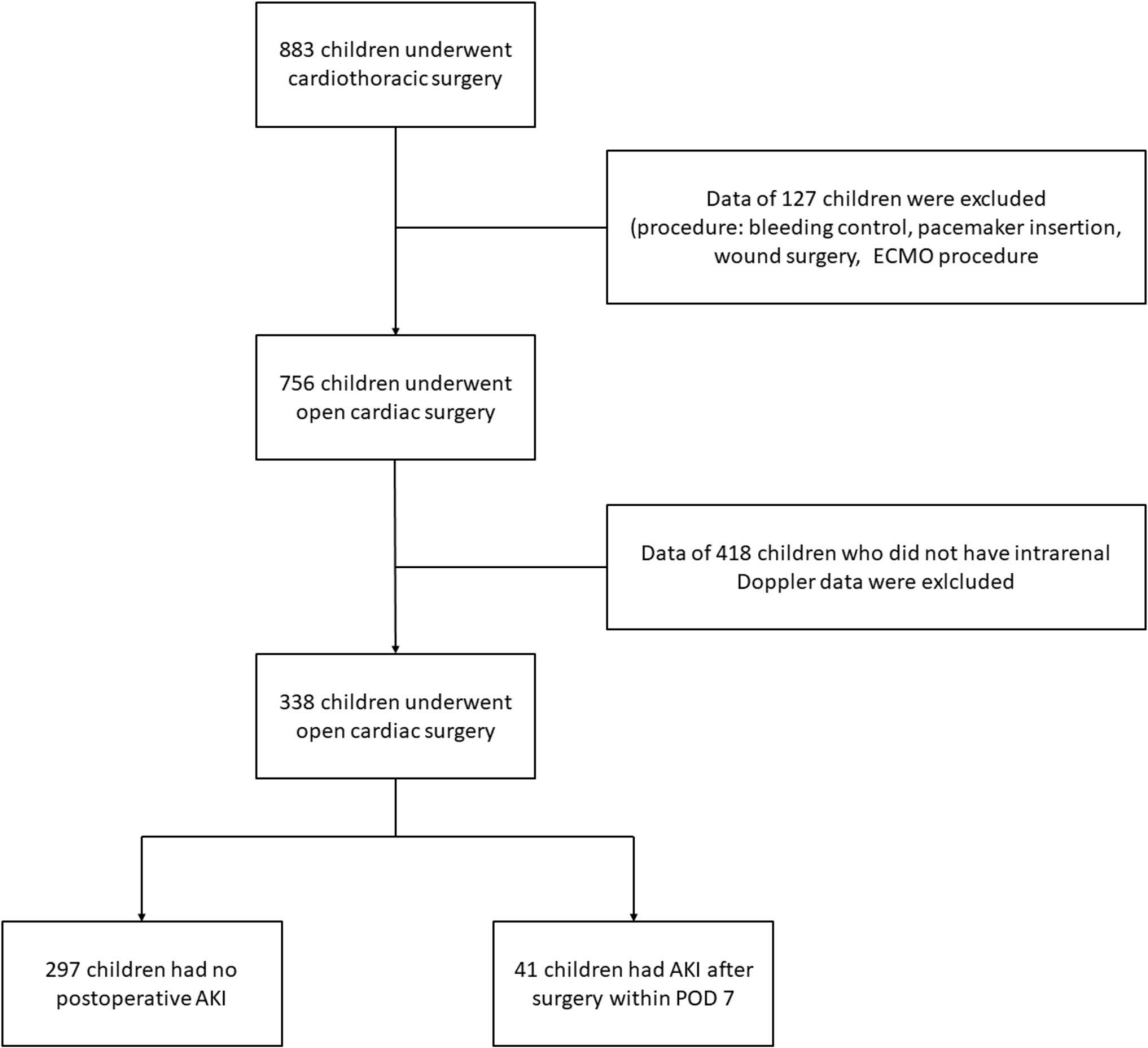
Flowchart of this retrospective study

Between 2019 and December 2021, 883 pediatric patients underwent cardiothoracic surgery at our center. Among them, 127 children who underwent bleeding control surgery (n = 22), pacemaker-related operations (*n* = 46), wound surgery (*n* = 28), or ECMO procedures (*n* = 31) were excluded. In total, 756 patients underwent open cardiac procedures. Renal vein and artery measurements were performed before and after CPB in 338 patients. Postoperative AKI occurred in 41 (12.1%) patients. Of these, 13 (31.7%) experienced grade 1 AKI, 2 (4.9%) developed grade 2 AKI, 26 (63.5%) progressed to grade 3 AKI (Fig. 2). All cases of AKI in our cohort were diagnosed within POD 4, and the overall median duration of AKI among affected patients was 6 days (IQR 4–7). The demographic and baseline variables are shown in Table 1. Patients with postoperative AKI were younger (median difference, −0.4; 95% confidence interval [CI], −1.0 to −0.2; *p* < 0.001) and had higher RACHS-1 category (odds ratio [OR], 4.3; 95% CI, 2.1–8.8; *p* < 0.001) than those without AKI.

**Table 1:** Baseline characteristics of children with or without postoperative acute kidney injury

Variables	Non-AKI (*n* = 297)	AKI (n = 41)	Effect size (95% CI)	*p* value
Age (year)	1.3 [0.9, 1.7; range 0.04–18]	0.2 [0.1, 0.6; range 0.01–18]	−0.4 (−1.0 to −0.2)	<0.001
Sex (M/F)	171 (57.6%)/126 (42.4%)	22 (53.7%)/19 (46.3%)	1.2 (0.6 to 2.3)	0.635
Height (cm)	78.5 [61.9, 102.2]	58.0 [51.1, 91.2]	−13.4 (−21.8 to −6.7)	<0.001
Weight (kg)	9.7 [6.1, 15.8]	4.9 [3.5, 13.3]	−3.1 (−5.1 to −1.6)	<0.001
RACHS-1 (low/high)*	179 (60.3%)/118 (39.7%)	11 (26.8%)/30 (73.2%)	4.3 (2.1 to 8.8)	<0.001
Anatomy				0.167
ASD/PFO	60 (20.2%)	5 (12.2%)		
VSD (with or without ASD)	66 (22.2%)	9 (22.0%)		
AVSD	33 (11.1%)	8 (19.5%)		
DORV with VSD	6 (2.0%)	0		
TOF/PA VSD with or without MAPCA	54 (18.2%)	4 (9.8%)		
RVOT stenosis/PS without other anomaly	8 (2.7%)	0		
Coarctation of aorta/aortic stenosis	13 (4.4%)	1 (2.4%)		
Hypoplastic left heart syndrome	12 (4.0%)	4 (9.8%)		
MR/TR	18 (6.1%)	2 (4.9%)		
Truncus arteriosus	9 (3.0%)	2 (4.9%)		
TGA	9 (3.0%)	2 (4.9%)		
TAPVC	5 (1.7%)	2 (4.9%)		
Anomalous of coronary artery origin	4 (1.3%)	0		

Data are expressed in the form of median [interquartile range] and n (%).

*AKI*, acute kidney injury; *CI*, confidence interval; *M*, male; *F*, female; *RACHS-1*, Risk adjustment for congenital heart surgery

Anatomy: *ASD*, atrial septal defect; *PFO*, patent foramen ovale; *VSD*, ventricular septal defect; *AVSD*, atrioventricular septal defect; *DORV*, double outlet right ventricle; *TOF*, tetralogy of Fallot; *PA*, pulmonary atresia; *MAPCA*, major aortopulmonary collateral arteries; *RVOT*, right ventricular outflow tract; *MR*, mitral regurgitation; *TR*, tricuspid regurgitation; *TGA*, transposition of great arteries; *TAPVC*, total anomalous pulmonary venous connection

^*^RACHS-1 score 1 and 2 were categorized as low grade, whereas 3 to 6 were categorized as high grade.

The intrarenal Doppler data of the children with and without AKI are presented in Table 2. No difference was found in the distribution of renal venous Doppler patterns between children with and those without AKI after anesthesia induction. However, after operation, children with AKI had more patterns 2 and 3 of renal venous Doppler than those without AKI (63.5% vs. 19.9%; mean difference, 43.5%; 95% CI, 27.2–59.9%; *p* < 0.001). Among 331 patients, 62 patients (18.7%) experienced a worsening in their renal venous Doppler pattern after surgery, whereas 269 (81.3%) either maintained or showed improvement in their Doppler pattern. Postoperative worsening of renal venous Doppler patterns was significantly associated with in increased likelihood of developing grade 2 and 3 AKI (OR = 1.9, 95% CI, 1.1–2.7; *p* < 0.001).

**Table 2:** Echocardiographic data before and after operative procedure in children with or without postoperative acute kidney injury

	Non-AKI (*n* = 297)	AKI (*n* = 41)	Effect size (95% CI)	*p* value
*Before the operative procedure*				
Ejection fraction^a^(%)	67.0 [61.4, 73.0]	67.0 [57.0, 71.5]	−1.5 (−5.0 to 2.0)	0.373
Fractional area change^b^ (%)	39.5 [32.3, 46.9]	38.1 [31.6, 43.1]	−1.0 (−4.2 to 0.7)	0.056
Renal resistive index	0.75 [0.66, 1.00]	1.00 [0.76, 1.00]	0.12 (0.02 to 0.19)	<0.001
Renal vein Doppler pattern				0.977
Pattern 1 Pattern 2 Pattern 3	215 (72.4%) 61 (20.5%) 21 (7.01%)	30 (73.2%) 8 (19.5%) 3 (7.3%)		
*After the operative procedure*				
Ejection fraction^a^(%)	61.0 ± 12.9	57.1 ± 11.8	−3.9 (−8.5 to 0.8)	0.101
Fractional area change^b^ (%)	45.8 [38.0, 51.7]	43.1 [32.1, 52.1]	−2.2 [−5.8 to 0.6]	0.180
Renal resistive index	0.76 [0.68, 0.87]	0.90 [0.79, 1.00]	0.11 (0.06 to 1.56)	<0.001
Renal vein Doppler pattern				<0.001
Pattern 1 Pattern 2 Pattern 3	237 (80.1%) 43 (14.5%) 16 (5.4%)	15 (36.6%) 17 (41.5%) 9 (22.0%)		

Data are expressed in the form of mean ± SD, median [interquartile range] and *n* (%).

*AKI*, acute kidney injury; *CI*, confidence interval; *EF*, ejection fraction; *RI*, resistive index

^a^Ejection fraction of systemic ventricle

^b^Function of right ventricle

Patients without postoperative AKI had lower median value of renal RI postoperatively (0.76 vs. 0.90; median difference, 0.11; 95% CI, 0.06–1.56; *p* < 0.001) and preoperatively (0.75 vs. 1.00; median difference, 0.12; 95% CI, 0.02–0.19; *p* < 0.001) compared to those with AKI.

The other preoperative and intraoperative variables are shown in Table 3. Compared with the non-AKI group, more patients in the AKI group had a history of ICU admission (2.7% vs. 31.7%; OR, 16.8; 95% CI, 6.4–43.9; *p* < 0.001), lower preoperative albumin level (4.3 vs. 3.9 g/dL; mean difference, −0.3 g/dL; 95% CI, −0.5 to −0.1 g/dL; *p* < 0.001), and lower GFR (73.3 vs. 59.9 mL/min/1.73 m^2^; median difference, −12.0 mL/min/1.73 m^2^; 95% CI, −17.5 to −6.3 mL/min/1.73 m^2^; *p* < 0.001). During the operation, the amount of transfusion was larger (median difference, 1.1; 95% CI, 0.0–2.1; *p* = 0.003) and maximum VIS value was higher (median difference, 7.0; 95% CI, 5.0–10.0; *p* < 0.001) in the AKI group than those in the non-AKI group. When the physiologic type after the operation was divided into bi-ventricular and single ventricular types, regardless of preoperative cardiac morphologic or physiologic type, the proportion of single ventricle type was higher in patients who developed AKI than that in those who did not develop AKI (12.5% vs. 26.8%; OR, 0.4; 95% CI, 0.2–0.8; *p* = 0.016).

**Table 3:** Preoperative, intraoperative and immediate postoperative variables in children with or without postoperative acute kidney injury

	Non-AKI (*n* = 297)	AKI (*n* = 41)	Effect size (95% CI)	*p* value
*Preoperative variables*				
MV care, *n* (%)	3 (1.0%)	3 (7.3%)		0.014
ICU admission, *n* (%)	8 (2.7%)	13 (31.7%)		<0.001
PHT, *n* (%)	83 (28.0%)	9 (22.0%)		0.420
Hemoglobin, g/dL	12.7 [11.6, 14.0]	13.6 [11.6, 14.4]	0.4 (−0.3 to 1.2)	0.218
Albumin, g/dL	4.3 ± 0.3	3.9 ± 0.5	−0.3 (−0.5 to −0.1)	<0.001
Creatinine, mg/dL	0.45 [0.41, 0.52]	0.46 [0.39, 0.49]	−0.01 (−0.04 to 0.02)	0.488
GFR, mL/min/1.73 m^2^	73.3 [61.9, 85.8]	59.5 [51.9, 75.5]	−12.0 (−17.5 to −6.3)	<0.001
Fibrinogen, mg/dL	223.0 [196.8, 256.0]	231.0 [179.3, 264.8]	−1.0 (−21.0 to 19.0)	0.929
*Intraoperative variables*				
Anesthesia time, hour	6.2 [5.3, 7.5]	6.8 [5.7, 8.5]	0.6 (0.0 to 1.2)	0.060
CPB time, min	147.1 ± 59.0	158.0 ± 103.3	10.9 (−10.5 to 32.3)	0.317
ACC time, min	90.2 ± 43.3	103.4 ± 54.1	13.2 (−8.2 to 29.7)	0.116
Transfusion, ml/kg/hr	1.0 [0.0, 3.4]	2.9 [0.9, 6.1]	1.1 (0.0 to 2.1)	0.003
Urine output, ml/kg/hr	3.5 [2.7, 4.5]	4.2 [2.9, 5.4]	0.4 (−0.2 to 1.0)	0.185
VIS	15.0 [7.0, 15.0]	20.0 [15.0, 29.3]	7.0 (5.0 to 10.0)	<0.001
Maximum glucose, mg/dL	202.2 ± 44.6	203.9 ± 59.2	1.7 (−13.5 to –16.9)	0.825
Repair type (BV/SV)	260 (87.5%)/37 (12.5%)	30 (73.2%)/11 (26.8%)		0.016

Data are expressed in the form of mean ± SD), median [interquartile range] and *n* (%).

*ACC*, aorta cross clamp; *AKI*, acute kidney injury; *BV*, bi-ventricular repair; *CI*, confidence interval; *CPB*, cardiopulmonary bypass; *CRP*, C-reactive protein; *EF*, ejection fraction; *GFR*, glomerular filtration rate; *ICU*, intensive care unit; *LOS*, length of stay; *MV*, mechanical ventilator; *PHT*, pulmonary hypertension; *RACHS*, Risk adjustment for congenital heart surgery; *RI*, resistive index; *SV*, single ventricular repair; *VIS*, vasoactive-inotropic score

**Fig. 3:**
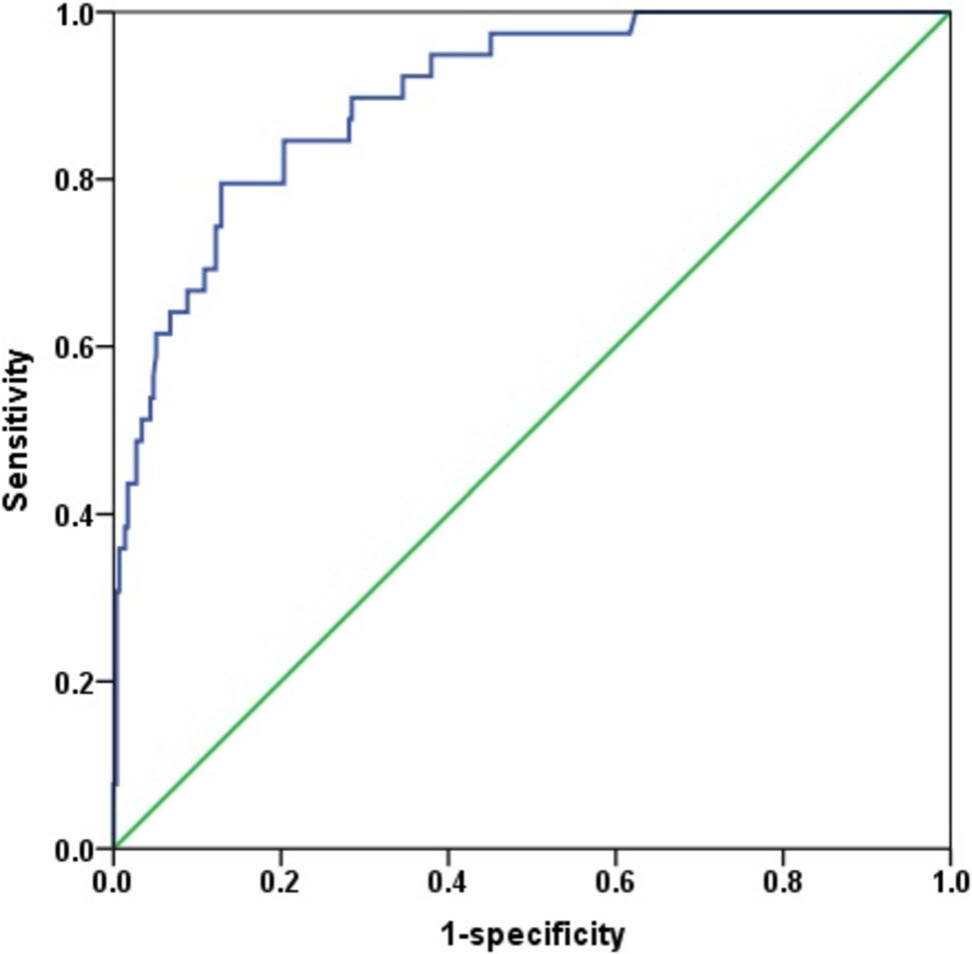
Receiver Operating Characteristic curve of the multivariate logistic regression model for predicting postoperative acute kidney injury (AKI). The model demonstrated excellent discriminative ability with an area under the curve of 0.890, indicating high sensitivity and specificity for predicting AKI in pediatric cardiac surgery patients

Age, RACHS-1 score, preoperative ICU admission, preoperative albumin level, preoperative GFR, duration of anesthesia, intraoperative transfusion, intraoperative VIS, repair type (single ventricular repair), preoperative and postoperative renal RI, and postoperative renal vein Doppler pattern yielded *p* ≤ 0.1 in univariate analysis. Those were included in the multivariate logistic regression analysis. Consequently, preoperative ICU admission, high RACHS-1 score, low preoperative albumin level, high intraoperative VIS level, and renal venous Doppler patterns 2 and 3 were associated with AKI in children after cardiac surgery (Table 4). This multivariate logistic regression model for postoperative AKI showed good discriminative ability (C-index=0.890, Fig. 3) and predictive power (93.0%), and the Hosmer–Lemeshow test showed a good fit (chi-squared test=5.516, *p* = 0.701).

**Table 4:** Multivariate analysis of associated factors including intrarenal venous Doppler patterns for postoperative acute kidney injury

	Multivariate analysis		
Variables	Odds ratio	95% CI	*p* value
Preop. ICU stay admission	7.1	2.1–23.4	0.001
RACHS-1 (high)	4.5	1.6–12.5	0.004
Preoperative albumin. g/dL	0.2	0.1–0.7	0.010
Intraoperative VIS	1.1	1.0–1.1	0.001
Intrarenal venous Doppler pattern			
Pattern 1	1.0 (Reference)		
Pattern 2	4.7	1.7–13.1	0.003
Pattern 3	10.2	3.3–31.8	<0.001

*CI*, confidence interval; *GFR*, glomerular filtration rate; *ICU*, intensive care unit; *RACHS*, Risk adjustment for congenital heart surgery; *VIS*, vasoactive-inotropic score

Additionally, a separate logistic regression analysis was performed, categorizing patients into no AKI/grade 1 AKI (*n* = 311) and grade 2 and 3 AKI (*n* = 27). The results showed that a higher RACHS-1 score (OR 4.9; 95% CI, 1.4–16.5; *p* = 0.011), preoperative ICU admission (OR 5.9; 95% CI, 1.6–22.2; *p* = 0.008), and higher intraoperative VIS (OR 1.1; 95% CI, 1.0–1.1; *p* = 0.002) were significantly associated with severe AKI. Furthermore, renal venous Doppler pattern 2 was associated with an 8.5-fold increased risk (95% CI, 2.6–27.6; *p* < 0.001), while renal venous Doppler pattern 3 showed a 6.7-fold increased risk (95% CI, 1.6–27.9; *p* = 0.01). In this model, the predictive power was 94.8%, with a C-index of 0.616. The Hosmer–Lemeshow test confirmed a good model fit (chi-squared test=7.394, *p* = 0.495).

## Discussion

In this study, we compared the intrarenal venous Doppler patterns measured intraoperatively between pediatric patients with and without AKI after cardiac surgery. We found that biphasic and monophasic renal venous flows were observed more frequently in children with postoperative AKI than in those without AKI. These intrarenal venous patterns were associated with the occurrence of AKI, and other factors, such as preoperative ICU admission, preoperative lower GFR, and single ventricle physiology after repair, were associated with AKI in children undergoing cardiac surgery.

Right ventricular (RV) dysfunction after pediatric cardiac surgery is critical [14]. It leads to venous congestion, and elevated central venous pressure can affect low-resistance renal vessels and rapidly increase interstitial pressure in the encapsulated kidney. This reduces the GFR and may result in AKI development [18, 19]. Renal Doppler ultrasonography is a valuable tool for assessing renal congestion [9, 11, 19]. The assessment of intrarenal venous flow patterns provides insights into renal venous congestion [11].

One of the key advantages of renal Doppler is its bedside applicability—it is noninvasive and fast and provides early detection of renal congestion—making it highly useful for perioperative monitoring. These benefits highlight the potential of Doppler ultrasonography as a tool for the early identification of patients at risk of AKI and for guiding timely interventions. Several studies on the usefulness of intrarenal venous flow examination for the detection of AKI have been published in the adult population [11]. However, pediatric data are limited, and this is the first study to assess the usefulness of renal Doppler measurements in identifying postoperative AKI in the pediatric population.

The reported incidence rate of AKI after pediatric cardiac surgery is wide, ranging between 11% and 42% [20–22]. According to a previous retrospective study conducted in our center, the incidence rate of AKI following cardiac surgery was approximately 36% [23]. In the present study, the incidence rate of AKI was 12.1%, which seemed to be an underestimate, as we only included children with available intrarenal artery and venous flow data.

In this study, a higher renal RI and more biphasic and monophasic intrarenal venous flow patterns were observed in children with AKI. When intrarenal resistance increases, the diastolic velocity of the renal arterial blood decreases, leading to an increase in the RI [24]. The normal range of the mean RI is 0.50–0.64 in adults [25]. However, the mean RI commonly exceeds 0.7 in infants, and this can also be observed up to 4 years of age [26]. Our patients showed a relatively high RI even before surgery. A relatively young median age and CHD likely contributed to high preoperative RI values. Preoperative conditions may have led to elevated renal vascular resistance, further explaining the higher RIs observed in our cohort.

According to the multivariate logistic regression model, not higher renal RI, but abnormal intrarenal venous Doppler patterns were associated with AKI, with high odds ratio, in this study. This result is concordant with those of previous studies, demonstrating that discontinuous renal venous flow is more strongly correlated with AKI and clinical outcomes than other Doppler parameters [9, 27]. Although renal RI is useful for detecting changes in renal vascular resistance, it is influenced by many factors beyond renal congestion, such as arterial stiffness, systemic blood pressure, and patients’ age [27]. However, the discontinuous renal venous Doppler pattern is known as a real-time impairment in venous outflow from the kidney, which is a hallmark of renal congestion [28].

In this study, we did not find a significant association between renal venous Doppler patterns and the AKI grade. This lack of association may be attributed to developmental differences in renal hemodynamics between children and adults. As the kidneys mature, hemodynamic patterns may occur, indicating that the variations observed in adult Doppler patterns may not have the same implications in pediatric populations. In young children, elevated renal vascular resistance and immaturity in autonomic blood flow regulation can lead to inconsistent Doppler flow patterns compared with adults [24, 29, 30]. Additionally, pediatric AKI can be caused by factors, such as anemia, hemoconcentration, inflammation, and hypoperfusion [31] and the impact of these factors on renal blood Doppler patterns remains unclear. However, based on the results of this study, we believe that renal congestion may be a significant risk factor for AKI in pediatric patients.

In addition to renal venous Doppler patterns, preoperative ICU admission, high RACHS-1 score and intraoperative VIS, and low preoperative albumin levels were associated with postoperative AKI. According to a meta-analysis, RACHS-1 score ≥3 and vasopressor use were the significantly and consistently associated factors of AKI [22]. In addition, pediatric patients presenting with preoperative uncontrolled hypoxia and symptoms of heart failure are typically admitted to the ICU prior to corrective cardiac surgery. These preoperative conditions can serve as significant risk factors for AKI development following cardiac surgery [22]. Regarding albumin level, hypoalbuminemia is associated with unfavorable outcomes after cardiac surgery [32]. In addition, we have previously found that high preoperative serum albumin levels may have protective effects against AKI in children [23].

This study had some limitations. First, as a retrospective study, it was subject to inherent biases and limitations in data availability. For example, we only included the data of patients who underwent intrarenal blood flow Doppler measurements, which might have introduced a selection bias. In addition, this selection bias could have contributed to the difference in AKI incidence compared with other studies and the small sample size. Second, a wide age range was included in this study, as normal renal venous patterns may vary with age. To accurately define pathologic features in Doppler parameters, continuous follow-up from one day to the next is necessary in the pediatric population [24]. Future studies should stratify patients based on age to better understand the renal venous patterns in different pediatric subpopulations. Third, we did not fully consider the use of nephrotoxic drugs known to influence AKI development [22]. Despite these limitations, our results suggest that routine intraoperative assessment of renal venous flow can aid in identifying children at a high risk of AKI, allowing for timely interventions to prevent or reduce renal injury.

In conclusion, we found a significant association between abnormal intrarenal venous Doppler patterns and occurrence of postoperative AKI in children undergoing cardiac surgery. Children with biphasic and monophasic renal venous flow patterns were at a high risk of developing AKI, and factors such as preoperative ICU admission, high RACHS-1 score, intraoperative VIS, and low preoperative albumin level were also identified as key risk factors. These findings emphasize the potential utility of routine intraoperative renal venous Doppler assessments for the early identification of patients at risk of AKI. Further studies are required to refine Doppler-based diagnostic protocols and explore their role in guiding timely interventions to improve postoperative outcomes.

## Data Availability

No datasets were generated or analysed during the current study.

## Abbreviations

AKI: Acute kidney injury
CPB: Cardiopulmonary bypass
ICU: Intensive care unit
CHD: Congenital heart disease
RI: Resistive index
RACHS-1: Risk Adjustment for Congenital Heart Surgery-1
GFR: Glomerular filtration rate
VIS: Vasoactive-inotropic score
MAEs: Major adverse events
ECMO: Extracorporeal membrane oxygenation
POD: Postoperative day
TTE: Transthoracic echocardiography
TEE: Transesophageal echocardiography
Vmax: Highest systolic blood velocity
Vmin: Lowest diastolic blood velocity
KDIGO: Kidney Disease Improving Global Outcomes
